# Transmission patterns of HIV-1 non-R5 strains in Poland

**DOI:** 10.1038/s41598-019-41407-7

**Published:** 2019-03-21

**Authors:** Joanna Smoleń-Dzirba, Magdalena Rosińska, Piotr Kruszyński, Janusz Janiec, Mariusz Cycoń, Jolanta Bratosiewicz-Wąsik, Marek Beniowski, Monika Bociąga-Jasik, Elżbieta Jabłonowska, Bartosz Szetela, Tomasz J. Wąsik

**Affiliations:** 1 Department of Microbiology and Virology, School of Pharmacy with the Division of Laboratory Medicine in Sosnowiec, Medical University of Silesia, Katowice, Poland; 2 Department of Infectious Disease Epidemiology and Surveillance, National Institute of Public Health - National Institute of Hygiene, Chocimska 24, Warsaw, Poland; 3 Department of Biopharmacy, School of Pharmacy with the Division of Laboratory Medicine in Sosnowiec, Medical University of Silesia, Katowice, Poland; 4 Out Patients Clinic for AIDS Diagnostics and Therapy, Specialistic Hospital in Chorzow, Zjednoczenia 10, Chorzów, Poland; 5 Department of Infectious Diseases, Jagiellonian University Medical College, Śniadeckich 5, Kraków, Poland; 6 Department of Infectious Diseases and Hepatology, Medical University of Lodz, Kniaziewicza 1, Łódź, Poland; 7 Department of Infectious Diseases, Hepatology and Acquired Immune Deficiencies, Wroclaw Medical University, Koszarowa 5, Wrocław, Poland

## Abstract

HIV-1 *env* sequencing enables predictions of viral coreceptor tropism and phylogenetic investigations of transmission events. The aim of the study was to estimate the contribution of non-R5 strains to the viral spread in Poland. Partial proviral *env* sequences were retrieved from baseline blood samples of patients with newly diagnosed HIV-1 infection between 2008–2014, including 46 patients with recent HIV-1 infection (RHI), and 246 individuals with long-term infection (LTHI). These sequences were subjected to the genotypic coreceptor tropism predictions and phylogenetic analyses to identify transmission clusters. Overall, 27 clusters with 57 sequences (19.5%) were detected, including 15 sequences (26.3%) from patients with RHI. The proportion of non-R5 strains among all study participants was 23.3% (68/292), and was comparable between patients with RHI and LTHI (11/46, 23.9% *vs* 57/246, 23.2%; p = 1.000). All 11 patients with non-R5 strains and RHI were men having sex with men (MSM). Among these patients, 4 had viral sequences grouped within phylogenetic cluster with another sequence of non-R5 strain obtained from patient with LTHI, indicating potential acquisition of non-R5 HIV-1 for at least 4/46 (8.7%) patients with RHI. We were unable to confirm the contribution of patients with RHI to the forward transmission of non-R5 strains, but a relatively high proportion of non-R5 strains among them deserves attention due to the limited susceptibility to CCR5 antagonists.

## Introduction

Human immunodeficiency virus type 1 (HIV-1) surface glycoprotein (gp120), a product of viral *env* gene, plays a key role in cell entry, viral tropism, pathogenesis, vulnerability to the host immune response and susceptibility to the entry inhibitors such as maraviroc^1–3^. The third variable loop (V3) of the gp120 contributes to the HIV-1 entrance into target cells by binding to the cell CD4 receptor followed by attachment to either the CCR5 or CXCR4 coreceptor molecules^1,4^. Viruses that during cell entry exclusively use the CCR5 or CXCR4 coreceptor are termed R5 or X4, respectively, whereas those that can use both coreceptors are described as R5X4 viruses. Usually, R5 viruses are associated with HIV-1 transmission and predominate at early phases of infection, while X4 or R5X4 (non-R5) viruses emerge as infection develops^1^, and are linked to accelerated disease progression and decline of CD4^+^ T-cell counts^5–7^. Although several mechanisms of selection against transmission of X4 viruses were proposed^8,9^, it has been reported that also CXCR4-using HIV-1 variants can be transmitted to a new host, e.g. to individuals lacking functional CCR5 receptor^10^. The proportion of infections with CXCR4-using strains investigated among patients with early infection stages, suggesting transmission of X4 variants, ranged from 3% to 26% depending on the population examined and method used to assess viral coreceptor tropism^5,11–20^.

To date, studies on HIV-1 coreceptor tropism in Poland were restricted to the northern geographic region and were performed among patients with long-term HIV-1 infection and experience of antiretroviral therapy^21^, and among treatment-naïve persons with a new HIV-1 diagnosis^22^. In the second group the overall prevalence of non-R5 strains was 15.5% or 27.8% depending on the interpretation criteria used in genotypic tropism test, and an increase in the frequency of non-R5 strains during the study period was observed, suggesting either increase of late HIV-1 diagnoses or putative transmission of CXCR4-using viruses in the local population^22^. Nevertheless, these studies did not allow to evaluate the rate of transmission of non-R5 strains, while this information would be useful for reasonable planning of treatment programs, especially in the context of the strong push to identify early HIV-1 infections, and the potential role of CCR5 antagonists in pre-exposure prophylaxis^23^.

The analysis of HIV-1 *env* gene sequences enables not only genotypic predictions of viral coreceptor tropism, but also allows for phylogenetic investigations aimed at detection of transmission clusters, which can facilitate the understanding of the epidemiological links. Thus, it is possible to study the contribution of patients with specified characteristics to HIV-1 transmission, and e.g. confirm the forward transmission of non-R5 strains or viral spread from patients at different infection stages^24–26^.

To obtain a more complete picture of the HIV-1 epidemics in Poland, analyses of *env* gene viral sequences from patients with new HIV-1 diagnosis established in the years 2008–2014 and known proportion of recent HIV-1 infections, were performed. Investigations included viral and host characteristics associated with coreceptor tropism and identified transmission clusters, in order to trace the spread of non-R5 HIV-1 strains and assess the contribution of recent infections to the Polish HIV-1 epidemic.

## Results

### Patients and virus characteristics

The characteristics of the 292 patients included in the analysis are presented in Table 1. All study participants were of Polish origin and were naïve for antiretroviral therapy. Among them there were 46 (15.8%) patients with recent HIV-1 infection (RHI), and 246 (84.2%) individuals with long-term HIV-1 infection (LTHI). Sexual contacts between men were the most frequently reported transmission route (199/292; 68.2%), with the proportion not significantly different in groups with dissimilar duration of infection (RHI: 35/46, 76.1% *vs* LTHI: 164/246, 66.7%; p = 0.232). Similarly, there were no statistical differences between patients with RHI and LTHI with respect to other transmission routes, sex, age, and CCR5 Δ32 genotype status. Although patients recruited in Chorzów predominated in the study (130/292; 44.5%), patients recruited in Wrocław constituted the most numerous group among the individuals with RHI (21/46; 45.7%; p = 0.007). A statistically significant difference between individuals with recent and long-term HIV-1 infection was observed for median baseline CD4^+^ T-lymphocyte count (RHI: 564 *vs* LTHI: 396 cells/µl; p = 0.002), and proportion of patients with higher CD4^+^ T-lymphocyte count at diagnosis (>500 cells/μl – RHI: 26/43, 60.5% *vs* LTHI: 79/229, 34.5%; p = 0.002). A baseline HIV-1 viral load, usually high at early infection stages, unexpectedly was significantly higher among patients with LTHI (RHI: 4.19 *vs* LTHI: 4.51 log copies/ml; p = 0.045) (Table 1). We note however, that the average delay between the diagnosis and the sample collection in our study was 32 days, which means that in many cases of recent infection we may have captured an already stabilised viral load, rather than the initial peak.

**Table 1:** Characteristics of the study participants diagnosed in the years 2008–2014 with HIV-1 *env* sequences available.

Characteristics	All patients	Patients with HIV-1 infection		*P*
		Recent	Long-term	
	n (%)	n (%)	n (%)	
**Number enrolled**	**292 (100**.**0)**	**46 (15**.**8)**	**246 (84**.**2)**	
**Sex** ^**a**^				
Female	25 (8.6)	3 (6.5)	22 (9.0)	0.777^b^
Male	265 (91.4)	43 (93.5)	222 (91.0)	
**Age at HIV-1 diagnosis** ^**a**^				
Median (interquartile range)	29 (25–35)	29 (24–33)	30 (25–35)	0.171^c^
<30	151 (52.1)	28 (60.9)	123 (50.4)	0.203^b^
≥30	139 (47.9)	18 (39.1)	121 (49.6)	
**City of HIV-1 diagnosis**				**0**.**027**^d^
Chorzów	130 (44.5)	17 (37.0)	113 (45.9)	0.332^e^
Kraków	41 (14.0)	3 (6.5)	38 (15.5)	0.163^e^
Łódź	39 (13.4)	5 (10.9)	34 (13.8)	0.813^e^
Wrocław	82 (28.1)	21 (45.7)	61 (24.8)	**0.007** ^**e**^
**Self-reported HIV-1 transmission route**				0.728^d^
Sex between men (MSM)	199 (68.2)	35 (76.1)	164 (66.7)	0.232^e^
Sex between women and men (HET)	48 (16.4)	5 (10.9)	43 (17.5)	0.385^e^
Sex between men or women and men (MSM/HET)	13 (4.5)	2 (4.3)	11 (4.5)	1.000^e^
Injecting drug use (IDU)^&^	20 (6.8)	3 (6.5)	17 (6.9)	1.000^e^
Other/Unknown	12 (4.1)	1 (2.2)	11 (4.5)	0.699^e^
**CCR5 Δ32 genotype status**				**0**.**039**^**d**^
wt/wt	228 (78.1)	38 (82.6)	190 (77.2)	0.560^e^
wt/Δ32	63 (21.6)	7 (15.2)	56 (22.8)	0.330^e^
Δ32/Δ32	1 (0.3)	1 (2.2)	—	0.158^e^
**HIV-1 viral load at diagnosis**, median log copies/ml (interquartile range)	4.47 (3.86–5.06)	4.19 (3.32–4.81)	4.51 (3.93–5.08)	**0**.**045**^**c**^
**CD4**^**+**^ **T-cell count at diagnosis**, median cells/µl (interquartile range)	411 (292.5–579) (n = 272)	564 (374–722) (n = 43)	396 (280–545) (n = 229)	**0**.**002**^**c**^
**No**. **of patients with CD4**^**+**^ **T-cell count at diagnosis**				**0**.**014**^**d**^
<200/µl	38 (14.0)	4 (9.3)	34 (14.8)	0.473^e^
200–349/µl	63 (23.2)	5 (11.6)	58 (25.3)	0.051^e^
350–499/µl	66 (24.3)	8 (18.6)	58 (25.3)	0.439^e^
>500/µl	105 (38.6)	26 (60.5)	79 (34.5)	**0**.**002**^**e**^

^a^for 2 individuals with long-term HIV-1 infection data on sex and age were not available, ^b^two-tailed Fisher’s exact test, ^c^Mann-Whitney *U* test, ^d^Pearson’s Chi-square test, ^e^two-tailed Fisher’s exact test comparing the specified category *vs* all other categories, ^&^includes persons who reported injecting drug use together with heterosexual intercourses (n = 11) or with sex between men (n = 1).

According to the subtype analysis of 292 HIV-1 *env* sequences, the majority of patients were infected with subtype B (279; 95.6%) (Table 2). Among 13 patients with non-B subtype infection, there were 8 individuals harbouring sub-subtype A6 (2.7%), 3 persons with CRF50_A1D infection, and remaining 2 persons were infected with CRF02_AG and sub-subtype F1, respectively. Strains of sub-subtype A6 were detected in all four diagnostic centres, and among patients who acquired HIV-1 infection either through same sex contact between men (n = 5) or heterosexual contact (n = 1) or via drug injections (n = 2), while CRF50_A1D strains were found exclusively in Kraków, among patients with homosexual (n = 2) or heterosexual (n = 1) route of transmission. CRF02_AG and F1 were identified in men who have sex with men (MSM) recruited in Kraków and Wrocław, respectively. There were no statistically significant differences regarding subtype frequency between patients with recent and long-term HIV-1 infection (p = 0.176). However, non-B subtypes were significantly less frequent among patients diagnosed in Chorzów than in other cities (Chorzów: 2/130, 1.5% *vs* other diagnostics centres: 11/162, 6.8% p = 0.043), and more frequent among patients diagnosed in Kraków (Kraków: 5/41, 12.2% *vs* other diagnostics centres: 8/251, 3.2% p = 0.023).

**Table 2:** Virus characteristics based on gp120 sequences obtained from the study participants in the years 2008–2014.

Characteristics	HIV-1 from all patients	HIV-1 from patients with infection		*P*
		Recent	Long-term	
	n (%)	n (%)	n (%)	
**Number enrolled**	**292 (100**.**0)**	**46 (15**.**8)**	**246 (84**.**2)**	
**HIV-1 subtype (based on gp120 sequences)**				0.176^a^
B	279 (95.6)	43 (93.5)	236 (95.9)	0.438^b^
A6	8 (2.7)	1 (2.2)	7 (2.9)	1.000^b^
CRF50_A1D	3 (1.0)	1 (2.2)	2 (0.8)	0.403^b^
CRF02_AG	1 (0.3)	—	1 (0.4)	1.000^b^
F1	1 (0.3)	1 (2.2)	—	0.158^b^
**Non-R5 strains according to:**				
Geno2pheno 10% FPR (subtype B) + PhenoSeq (non-B subtypes)	68 (23.3)	11 (23.9)	57 (23.2)	1.000^c^
Geno2pheno				
5.75% FPR	28 (9.6)	4 (8.7)	24 (9.8)	1.000^c^
10% FPR	68 (23.3)	11 (23.9)	57 (23.2)	1.000^c^
15% FPR	102 (34.9)	18 (39.1)	84 (34.2)	0.506^c^
20% FPR	133 (45.5)	23 (50.0)	110 (44.7)	0.523^c^
**Geno2pheno %FPR** - median (interquartile range)	23.5 (10.5–49.8)	19.5 (10.5–55.7)	23.7 (10.5–49)	0.578^d^
**Frequency of ambiguous nucleotides in** ***env*** **sequences, %** - median (interquartile range)	0 (0–0.28)	0 (0–0)	0.14 (0–0.41)	**<0.001** ^**d**^

^a^Pearson’s Chi-square test, ^b^two-tailed Fisher’s exact test comparing the specified category *vs* all other categories, ^c^two-tailed Fisher’s exact test, ^d^Mann-Whitney *U* test.

The median frequency of ambiguous nucleotides in the analysed *env* sequences was significantly lower among patients with RHI than those with LTHI (RHI: 0% (0–0) *vs* LTHI: 0.14% (0–0.41); p < 0.001) (Table 2).

### Coreceptor usage

The frequency of non-R5 strains among all patients included in the study was 23.3% (68/292) when the geno2pheno 10% FPR cut-off was applied for the subtype B sequences and PhenoSeq results were used for non-B subtypes (Table 2). The same frequency of non-R5 variants was observed for all sequences analysed with geno2pheno 10% FPR only. Applying geno2pheno FPR cut-off values of 15% and 20% resulted in the frequency of non-R5 strains as high as 34.9% (102/292) and 45.5% (133/292), respectively. With the more restrictive 5.75% FPR cut-off the frequency of non-R5 strains was decreased to 9.6% (28/292). There were no significant differences between persons with RHI and LTHI regarding the frequency of the non-R5 strains, and values of geno2pheno FPRs were comparable between patients with recent and long-term HIV-1 infection (p = 0.578) (Table 2).

Comparison of all patients harbouring non-R5 and R5 strains predicted by geno2pheno 10% FPR for the subtype B sequences and PhenoSeq for non-B subtypes revealed no significant differences with regard to sex, age, city of diagnosis, CCR5 Δ32 genotype, median viral load, and HIV-1 subtype (Table 3). Similarly, no significant differences were seen between non-R5 and R5 strains with respect to the number of ambiguous nucleotides and deduced ambiguous amino acids in V3 coding region. When the same comparisons were performed exclusively among persons with RHI, again the lack of significant differences between non-R5 and R5 strains was observed. A trend toward a higher proportion of MSM among patients with non-R5 strains in comparison with those harbouring R5 strains, was observed when all samples were tested (p = 0.054). This trend reached the level of statistical significance when analysis was restricted to the samples obtained from patients with recent HIV-1 infection only (p = 0.044) (Table 3).

**Table 3:** Comparison of patients harbouring non-R5 and R5 strains predicted by V3 analysis with geno2pheno 10% FPR (for subtype B) and PhenoSeq (for non-B subtypes) algorithms.

Patient and virus characteristics	All patients with HIV-1 strains		*P*	Patients with recent infection (n = 46) with HIV-1 strains		*P*
	Non-R5 (%) N = 68 (23.3%)	R5 (%) N = 224 (76.7%)		Non-R5 (%) N = 11 (23.9%)	R5 (%) N = 35 (76.1%)	
**Sex** ^**a**^						
Female	5 (7.4)	20 (9.0)	0.808^b^	0	3 (8.6)	0.569^b^
Male	63 (92.7)	202 (91.0)		11 (100.0)	32 (91.4)	
**Age at HIV-1 diagnosis** ^**a**^						
Median (interquartile range)	29 (26–34)	29 (25–35)	0.981^c^	28 (22–32)	29 (25–34)	0.699^c^
<30	37 (54.4)	114 (51.4)	0.679^b^	7 (63.6)	21 (60.0)	1.000^b^
≥30	31 (45.6)	108 (48.6)		4 (36.4)	14 (40.0)	
**City of HIV-1 diagnosis**			0.408^d^			0.184^d^
Chorzów	35 (51.5)	95 (42.4)	0.211^e^	7 (63.6)	10 (28.6)	0.070^e^
Kraków	9 (13.2)	32 (14.3)	1.000^e^	0	3 (8.6)	0.569^e^
Łódź	10 (14.7)	29 (12.9)	0.688^e^	1 (9.1)	4 (11.4)	1.000^e^
Wrocław	14 (20.6)	68 (30.4)	0.126^e^	3 (27.3)	18 (51.4)	0.188^e^
**Self-reported HIV-1 transmission route**			0.342^d^			0.337^d^
Sex between men (MSM)	53 (77.9)	146 (65.2)	0.054^e^	11 (100.0)	24 (68.6)	**0**.**044**^**e**^
Sex between women and men (HET)	8 (11.8)	40 (17.9)	0.268^e^	0	5 (14.3)	0.317^e^
Sex between men or women and men (MSM/HET)	2 (2.9)	11 (4.9)	0.739^e^	0	2 (5.7)	1.000^e^
Injecting drug use (IDU)	4 (5.9)	16 (7.1)	1.000^e^	0	3 (8.6)	0.569^e^
Other/Unknown	1 (1.5)	11 (4.9)	0.307^e^	0	1 (2.9)	1.000^e^
**CCR5 Δ32 genotype status**			0.135^d^			0.179^d^
wt/wt	50 (73.5)	178 (79.5)	0.317^e^	8 (72.7)	30 (85.7)	0.374^e^
wt/Δ32	17 (25.0)	46 (20.5)	0.501^e^	2 (18.2)	5 (14.3)	1.000^e^
Δ32/Δ32	1 (1.5)	—	0.233^e^	1 (9.1)	0	0.239^e^
**HIV-1 viral load at diagnosis**, median log copies/ml (interquartile range)	4.48 (3.74–5.01)	4.47 (3.87–5.07)	0.526^c^	4.39 (3.74–4.80)	4.17 (3.26–4.82)	0.988^c^
**CD4**^**+**^ **T-cell count at diagnosis**, median cells/µl (interquartile range)	349.5 (202–604) (n = 66)	432.5 (316–571) (n = 206)	0.066^c^	651 (279–766)	550.5 (385.5–662.5) (n = 32)	0.513^c^
**No**. **of patients with CD4**^**+**^ **T-cell count at diagnosis**			**0.027** ^**d**^			0.731^d^
<200/µl	16 (24.2)	22 (10.7)	**0.008** ^**e**^	1 (9.1)	3 (9.4)	1.000^e^
200–349/µl	17 (25.8)	46 (22.3)	0.616^e^	2 (18.2)	3 (9.4)	0.589^e^
350–499/µl	12 (18.2)	54 (26.2)	0.248^e^	1 (9.1)	7 (21.9)	0.656^e^
>500/µl	21 (31.8)	84 (40.8)	0.245^e^	7 (63.6)	19 (59.4)	1.000^e^
**HIV-1 subtype**			0.858^d^			0.799^d^
B	66 (97.1)	213 (95.1)	0.739^b^	11 (100.0)	32 (91.4)	0.569^b^
Non-B	2 (2.9)	11 (4.9)		0	3 (8.6)	
**No**. **of ambiguous nucleotides in V3 coding region** (mean ± standard deviation)	0.5 ± 1.3	0.4 ± 1.0	0.793^c^	0	0.03 ± 0.2	0.610^c^
**No**. **of ambiguous amino acids in V3** (mean ± standard deviation)	0.4 ± 1.1	0.3 ± 0.9	0.716^c^	0	0	—

^a^for 2 individuals with R5 strain infection data on sex and age were not available, ^b^two-tailed Fisher’s exact test, ^c^Mann-Whitney *U* test, ^d^Pearson’s Chi-square test, ^e^two-tailed Fisher’s exact test comparing the specified category *vs* all other categories.

### Transmission clusters

**Figure 1:**
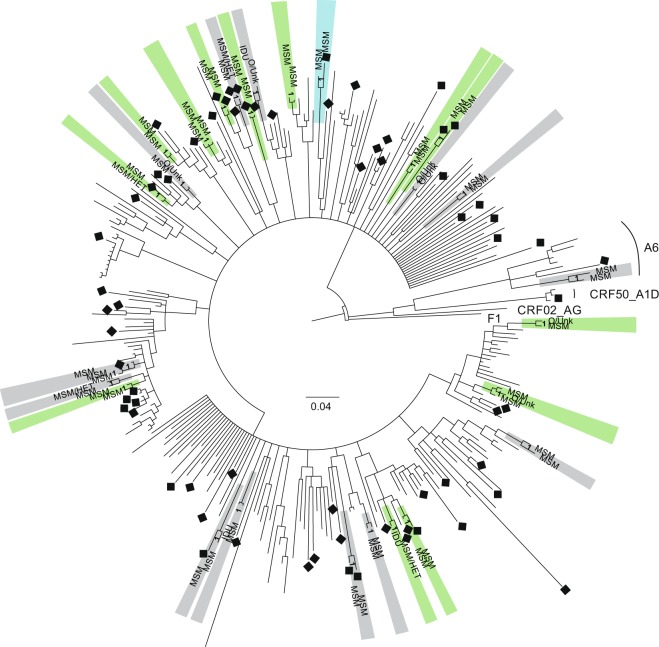
MCMC phylogenetic tree of *env* sequences obtained from 292 patients diagnosed in Poland in the years 2008–2014. Transmission clusters identified with the maximum likelihood aLRT value of >90%, maximum intracluster pairwise genetic distance <3%, and posterior probability of 1 in Bayesian inference are highlighted. Clusters highlighted in green contain sequences obtained from patients with long-term and recent HIV-1 infection. Clusters highlighted in grey and light blue contain sequences obtained from patients with long-term HIV-1 infection only and recent HIV-1 infection only, respectively. Squares indicate the presence of non-R5 strains. Self-reported transmission routes for patients with clustered HIV-1 sequences are specified with MSM (for sex between men), MSM/HET (for sex between men or women and men), IDU (for injecting drug use), O/Unk (for other/unknown). Majority of sequences represented subtype B, thus only non-B subtypes are indicated.

Among 292 HIV-1 sequences there were 27 transmission clusters containing 57 sequences (19.5%) identified (Table 4, Fig. 1). These clusters included from 2 to 3 sequences, with the majority of clusters containing only 2 sequences (24/27, 88.9%). The differences between patients with clustered and non-clustered HIV-1 sequences regarding the proportion of individuals of different sex, age, city of HIV-1 diagnosis, CCR5 Δ32 genotype, or infected with different HIV-1 subtypes or strains of different tropism were not significant, although a trend toward a higher prevalence of men within the group of patients with clustered sequences was noticed (p = 0.058) (Table 4). Patients with heterosexual transmission route were exclusively infected with viruses of non-clustered *env* sequences (p < 0.001), while patients for whom the route of transmission was unknown were more frequently represented among individuals with clustered HIV-1 sequences than among those with non-clustered sequences (p = 0.015). Similarly, higher proportion of MSM was observed within the group of clustered HIV-1 sequences, but the difference was not statistically significant (clustered: 44/57, 77.2% *vs* non-clustered: 155/235, 66.0%; p = 0.115). While the majority of clustered HIV-1 sequences were obtained from patients with LTHI (42/57, 73.7%), the proportion of sequences from persons with RHI was higher among clustered sequences than among non-clustered sequences (clustered: 15/57, 26.3% *vs* non-clustered: 31/235, 13.2%; p = 0.024).

**Table 4:** Comparison of patients with clustered and non-clustered HIV-1 *env* sequences and characteristics of clusters identified among 292 HIV-1 sequences.

Characteristics	Patients with HIV-1 sequences		*P*	Clustered sequences (n = 57) and clusters (n = 27) with				*P*
	clustered (%)*	non-clustered (%)		the same characteristics		different characteristics (mixed)		
	N = 57 (19.5%)	N = 235 (80.5%)		no. of sequences, %	no. of clusters	no. of sequences, %	no. of clusters	
**Sex** ^**a**^								
Female	1 (1.8)	24 (10.2)	0.058^b^	0	0	1 (1.8)	1	**0**.**036**^**b**^
Male	54 (98.2)	211 (89.8)		53 (96.4)	25	1 (1.8)		
**Age at HIV-1 diagnosis** ^**a**^								
Median (interquartile range)	29 (23–35)	29 (25–35)	0.414^c^					
<30	30 (54.5)	121 (51.5)	0.765^b^	18 (32.7)	9	12 (21.8)	11	0.595^b^
≥30	25 (45.5)	114 (48.5)		13 (23.6)	6	12 (21.8)		
**City of HIV-1 diagnosis**			0.907^d^					0.728^d^
Chorzów	27 (47.4)	103 (43.8)	0.658^e^	23 (40.4)	11	4 (7.0)	4	1.000^e^
Kraków	8 (14.0)	33 (14.0)	1.000^e^	7 (12.3)	3	1 (1.8)		1.000^e^
Łódź	6 (10.5)	33 (14.0)	0.664^e^	6 (10.5)	3	0		0.580^e^
Wrocław	16 (28.1)	66 (28.1)	1.000^e^	13 (22.8)	6	3 (5.3)		0.674^e^
**Self-reported HIV-1 transmission route**			**<0**.**001**^**d**^					**<0**.**001**^**d**^
Sex between men (MSM)	44 (77.2)	155 (66.0)	0.115^e^	36 (63.2)	17	8 (14.0)	9	**<0**.**001**^**e**^
Sex between women and men (HET)	0	48 (20.4)	**<0**.**001**^**e**^	—	—	—		—
Sex between men or women and men (MSM/HET)	4 (7.0)	9 (3.8)	0.290^e^	0	0	4 (7.0)		**0**.**010**^**e**^
Injecting drug use (IDU)	3 (5.3)	17 (7.2)	0.774^e^	0	0	3 (5.3)		**0**.**033**^**e**^
Other/Unknown	6 (10.5)	6 (2.6)	**0**.**015**^**e**^	2 (3.5)	1	4 (7.0)		0.088^e^
**CCR5 Δ32 genotype status**			0.879^d^					
wt/wt	45 (78.9)	183 (77.9)	1.000^e^					
wt/Δ32	12 (21.1)	51 (21.7)	1.000^e^					
Δ32/Δ32	0	1 (0.4)	1.000^e^					
**HIV-1 viral load at diagnosis**, median log copies/ml (interquartile range)	4.26 (3.67–4.95)	4.51 (3.89–5.07)	0.118^c^					
**CD4**^**+**^ **T-cell count at diagnosis**, median cells/µl (interquartile range)	502 (323.5–605.5) (n = 52)	399 (281.5–568) (n = 220)	**0**.**041**^**c**^					
**No**. **of patients with CD4**^**+**^ **T-cell count at diagnosis**			**0**.**014**^**d**^					
<200/µl	2 (3.8)	36 (16.4)	**0.024** ^**e**^					
200–349/µl	15 (28.8)	48 (21.8)	0.278^e^					
350–499/µl	8 (15.4)	58 (26.4)	0.108^e^					
>500/µl	27 (51.9)	78 (35.5)	**0**.**039**^**e**^					
**HIV-1 subtype**			0.849^d^					
B	55 (96.5)	224 (95.3)	1.000^b^					
Non-B	2 (3.5)	11 (4.7)						
**HIV-1 tropism** ^**f**^								
Non-R5 strains	16 (28.1)	52 (22.1)	0.383^b^	12 (21.1)	6	4 (7.0)	4	0.250^b^
R5 strains	41 (71.9)	183 (77.9)		36 (63.2)	17	5 (8.8)		
**HIV-1 infection status**								
Recent	15 (26.3)	31 (13.2)	**0**.**024**^**b**^	2 (3.5)	1	13 (22.8)	13	**<0**.**001**^**b**^
Long-term	42 (73.7)	204 (86.8)		27 (47.4)	13	15 (26.3)		

*clusters were selected based on aLRT value in ML method >0.9, maximum within cluster pairwise genetic distance threshold of 3%, and posterior probability in a Bayesian inference = 1, ^a^for 2 individuals with clustered HIV-sequences data on sex and age were not available, ^b^two-tailed Fisher’s exact test, ^c^Mann-Whitney *U* test, ^d^Pearson’s Chi-square test, ^e^two-tailed Fisher’s exact test comparing the specified category *vs* all other categories, ^f^according to geno2pheno 10% FPR (subtype B) + PhenoSeq (non-B subtypes).

The levels of baseline median viral load were comparable between patients infected with HIV-1 strains of clustered and non-clustered sequences. The median CD4^+^ T-cell count at diagnosis was significantly higher among patients from whom clustered HIV-1 sequences were obtained than among persons with non-clustered viral sequences (clustered: 502 *vs* non-clustered: 399; p = 0.041), and accordingly, the proportion of patients with CD4^+^ T-cell counts of <200/µl was significantly lower among those harbouring HIV-1 with clustered sequences, while the proportion of patients with >500 CD4^+^ T-cell counts among them was significantly higher (Table 4).

**Figure 2:**
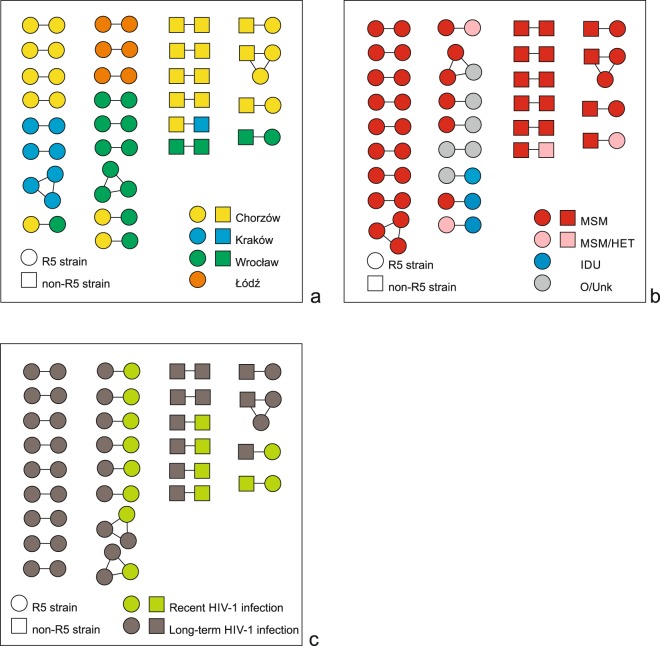
HIV-1 transmission clusters inferred from the analysis of *env* sequences obtained from 292 study participants diagnosed in the years 2008–2014. Clusters were identified with the maximum likelihood aLRT value of >90%, maximum intracluster pairwise genetic distance <3%, and posterior probability of 1 in Bayesian inference. Viral tropism is indicated with shapes: squares and circles represent sequences of non-R5 and R5 strains, respectively. For all patients with clustering viral sequences shapes are colored according to: city of HIV-1 diagnosis (**a**), self-reported transmission route (MSM - sex between men, MSM/HET - sex between men or women and men, IDU - injecting drug use, O/Unk - other/unknown) (**b**), and duration of HIV-1 infection (**c**).

To give a more detailed description of the 57 patients harbouring HIV-1 with clustered sequences and putatively engaged in transmission events, the numbers of clusters gathering HIV-1 sequences from patients of the same characteristics (sex, age group, city of diagnosis, transmission route, non-R5 strain infection, HIV-1 infection status), as well as the numbers of sequences in such clusters, were calculated (Table 4). Since the majority of clustered HIV-1 sequences were obtained from men, also the majority of clusters contained exclusively sequences obtained from men (25 clusters with 53 sequences), and only a single cluster contained one sequence from a woman and another from a man. Nine clusters included 18 HIV-1 sequences from persons under 30 years old only, 6 clusters contained 13 HIV-1 sequences from persons ≥30 years old only, and 11 clusters gathered 24 sequences from patients of <30 and ≥30 together. One cluster contained 2 sequences from persons for whom the information on sex and age was unavailable. The majority of clusters included sequences obtained from patients diagnosed with HIV-1 in the same location (23 clusters with 49 sequences). In the 4 remaining clusters, sequences obtained from patients recruited in different centres were grouped; within each of these 4 clusters there was 1 sequences from patient diagnosed in Chorzów combined either with sequence from patient diagnosed in Wrocław (3 clusters) or in Kraków (1 cluster) (Table 4, Fig. 2a).

The majority of clusters included sequences from persons who declared the same route of HIV-1 transmission, namely 17 clusters contained 36 sequences obtained exclusively from MSM, and 1 cluster gathered 2 sequences from persons with unknown transmission route (Table 4, Figs 1 and 2b). Among the remaining 9 mixed clusters, all but one contained at least one sequence from an MSM or MSM who also reported sex with women. Three mixed clusters included sequences from injecting drug users (IDUs), but none of these 3 sequences came from a person with RHI.

Most of clustered HIV-1 sequences obtained from patients with LTHI (27/42) were grouped within 13 clusters gathering only the sequences from patients with LTHI (Table 4, Figs 1 and 2c). The next 13 clusters contained sequences from both, persons with LTHI (15 sequences) and patients with RHI (13 sequences). There was only one cluster with 2 sequences from patients with RHI only.

To infer about possible transmission events from patients with recent HIV-1 infection we examined the sample collection dates for persons with viral sequences gathered in a single transmission cluster. These dates were checked for all 14 clusters containing 15 sequences obtained from patients with RHI. Onward transmission during the recency period could not be the case for any of the 2 patients with RHI, whose viral sequences were grouped in a single cluster, since one patient was recruited to the study over 4 years earlier than the second one, and therefore could be engaged in transmission only during long-standing HIV-1 infection. For 10 out of 13 mixed clusters, i.e. containing sequences from persons with RHI and LTHI, the date of sample collection for a patient with RHI was less than 6 months earlier than the sampling date for any patient with LTHI involved in the same cluster, thus we were not able to unambiguously demonstrate that these patients with recent HIV-1 infection were engaged in forward transmission.

As many as 23 clusters contained sequences of HIV-1 strains of the same tropism, among them there were 17 clusters with 36 sequences of R5 strains, and 6 clusters with 12 sequences of non-R5 variants. The remaining 9 clustered sequences were included within 4 mixed clusters, with sequences of R5 and non-R5 strains gathered simultaneously in each cluster (Table 4, Figs 1 and 2). Non-R5 strains were identified among 11 out of 46 patients with RHI, which may suggest acquisition of non-R5 variants for these patients. Among these 11 patients there were 5 with viral sequences grouped within 5 separate transmission clusters, and 6 with non-clustered sequences. More detailed characteristic of all patients with putative acquired non-R5 strains infection is presented in Table 5. Briefly, patients with possible acquired non-R5 infection were MSM with HIV-1 subtype B, most of them (7/11) were diagnosed in Chorzów. CCR5 genotypes wt/Δ32 and Δ32/Δ32 were identified in 3 of 11 patients (27.3%) with putative acquired non-R5 strains infection, and were not significantly more frequent than among patients with RHI harbouring R5 strains (5/35, 14.3%; p = 0.374). Among 5 clustered sequences obtained from patients with recent HIV-1 infection and non-R5 strains, there were 4 that grouped with non-R5 strains, further supporting potential acquisition of non-R5 strains for 4 out of 46 patients with RHI (8.7%) (Table 5).

**Table 5:** Possible acquired non-R5 strains infections among patients with clustered and non-clustered HIV-1 sequences.

Cluster	Patient	Sex	Age at HIV-1 diagnosis	City of HIV-1 diagnosis	Self-reported HIV-1 transmission route	HIV-1 infection status	Month and year of sample collection	CCR5 Δ32 genotype status	HIV-1 subtype	Viral tropism according to geno2pheno (FPR%)
Yes 2*	6938 MNS	Male	21	Chorzów	MSM	Recent	May 2009	wt/Δ32	B	**Non-R5 (6**.**9)**
	7051 KKS^a^	Male	29	Chorzów	MSM	Long-term	June 2009	wt/wt	B	**Non-R5 (6**.**7)**
Yes 5*	6903 TRS	Male	26	Chorzów	MSM	Recent	April 2009	wt/wt	B	**Non-R5 (1**.**7)**
	40 KOK^a^	Male	33	Kraków	MSM	Long-term	January 2010	wt/wt	B	**Non-R5 (1**.**7)**
Yes 7*	17 RKD	Male	32	Wrocław	MSM	Recent	September 2012	wt/wt	B	**Non-R5 (9**.**6)**
	19 MSD^a^	Male	26	Wrocław	MSM	Long-term	September 2012	wt/wt	B	**Non-R5 (9**.**6)**
Yes 8*	7987 LSS	Male	21	Chorzów	MSM	Recent	October 2010	wt/wt	B	**Non-R5 (7**.**4)**
	7019 KGS^a^	Male	23	Chorzów	MSM	Long-term	September 2009	wt/wt	B	**Non-R5 (7**.**4)**
Yes 9*	53 SCD	Male	29	Wrocław	MSM	Recent	September 2013	wt/Δ32	B	**Non-R5 (6**.**9)**
	20 KPD^a^	Male	37	Wrocław	MSM	Recent	April 2009	wt/wt	B	R5 (46.2)
No*	610 LJE	Male	37	Łódź	MSM	Recent	June 2008	wt/wt	B	**Non-R5 (6**.**0)**
No*	6571 JJ	Male	44	Chorzów	MSM	Recent	September 2008	wt/wt	B	**Non-R5 (3**.**9)**
No*	7595 PSS	Male	30	Chorzów	MSM	Recent	June 2010	wt/wt	B	**Non-R5 (7**.**1)**
No*	8031 RFS	Male	28	Chorzów	MSM	Recent	November 2010	wt/wt	B	**Non-R5 (8**.**2)**
No*	10055 DCS	Male	22	Chorzów	MSM	Recent	March 2013	Δ32/Δ32	B	**Non-R5 (1**.**7)**
No*	57 SSD	Male	24	Wrocław	MSM	Recent	February 2014	wt/wt	B	**Non-R5 (0**.**1)**

*data of patients with possibly acquired non-R5 strain infection, ^a^data of the partner involved in the same cluster (possible source of infection), MSM – sex between men, FPR – false positive rate.

### Time trends

The proportion of patients diagnosed during recent HIV-1 infection has increased significantly from 9.9% (7/71) in 2008 to 23.6% (13/55) in 2013 and 25% (1/4) in 2014 with an average annual difference of +3.15% per year (OR: 1.26, 95% CI: 1.07–1.48, p = 0.005) (Table 6). Consistently, there were significantly increasing mean CD4^+^ T-cell counts from 460 ± 249 in 2008 to 550 ± 354 in 2013 (slope =+22.1/year, p = 0.008). Significantly increasing trend in the CD4^+^ T-cell counts was not visible, when the analysis was restricted to patients with RHI (p = 0.286), but was true for the group of patients with LTHI (slope =+18.0/year, p = 0.047). Analysis of the viral load revealed no significant time trends, neither for all patients nor for patients with RHI or LTHI, separately.

**Table 6:** The logistic and linear regression analyses of time trends in selected parameters for patients with recent and long-term HIV-1 infection – years 2008–2014.

Patient and virus characteristics^a^	All patients			Patients with recent HIV-1 infection			Patients with long-term HIV-1 infection		
	OR (95%CI)	*P*	average annual difference	OR (95%CI)	*P*	average annual difference	OR (95%CI)	*P*	average annual difference
Recent HIV-1 infection	1.26 (1.07–1.48)	**0**.**005**	+3.15%						
Female	0.68 (0.52–0.91)	**0**.**003**	−2.38%	ND	ND	ND	0.75 (0.56–1.00)	**0**.**033**	−1.96%
Self-reported HIV-1 transmission route									
MSM	1.43 (1.23–1.66)	<**0**.**001**	+6.80%	1.23 (0.86–1.75)	0.245	+3.75%	1.46 (1.23–1.73)	<**0**.**001**	+7.30%
HET	0.61 (0.49–0.77)	<**0**.**001**	−5.10%	0.72 (0.43–1.21)	0.191	−3.05%	0.60 (0.46–0.78)	<**0**.**001**	−5.43%
IDU	0.89 (0.69–1.14)	0.340	−0.72%	1.14 (0.61–2.14)	0.667	+0.79%	0.84 (0.62–1.12)	0.213	−1.04%
Non-R5 strains^b^	0.92 (0.79–1.06)	0.234	−1.52%	0.89 (0.63–1.27)	0.528	−2.04%	0.92 (0.78-1.08)	0.290	−1.50%
Clustered HIV-1 transmission (3% genetic distance threshold)	1.04 (0.89-1.21)	0.634	+0.58%	0.75 (0.53-1.04)	0.074	−6.30%	1.10 (0.93–1.31)	0.283	+1.39%
HIV-1 non-B subtype	1.12 (0.84–1.48)	0.445	+0.49%	1.14 (0.61–2.14)	0.667	+0.79%	1.09 (0.79–1.51)	0.610	+0.35%
	**slope/year (95%CI)**	***P***		**slope/year (95%CI)**	***P***		**slope/year (95%CI)**	***P***	
Age at HIV-1 diagnosis, years	−0.41 (−0.91–0.09)	0.108		−0.50 (−1.58–0.58)	0.357		−0.33 (−0.90–0.25)	0.263	
HIV-1 viral load at diagnosis, log copies/ml	−4.03 (−4.59–4.24)	0.455		+4.22 (−4.63–4.88)	0.572		−4.20 (−4.68–4.22)	0.335	
CD4^+^ T-cell count at diagnosis, cells/μl	+22.1 (5.9–38.3)	**0**.**008**		+23.2 (−20.0–66.6)	0.286		+18.0 (0.2–35.8)	**0**.**047**	
Geno2pheno %FPR	+0.64 (−0.87–2.14)	0.404		+0.52 (−3.20–4.24)	0.780		+0.77 (−0.91–2.46)	0.368	

^a^in the first column outcome variables are specified, the year of HIV-1 diagnosis was a single predictor in the logistic and linear regression analyses, ^b^according to geno2pheno 10% FPR (subtype B) + PhenoSeq (non-B subtypes), ND – not determined, MSM – sex between men, HET – sex between women and men, IDU – injecting drug use, FPR – false positive rate.

In line with the decreasing proportion of women among HIV-1 infections (from 11.3%, 8/71 in 2008 to 0% in 2013 and 2014; average annual difference = −2.38%/year, OR: 0.68, 95% CI: 0.52–0.91, p = 0.003), the proportion of MSM has increased significantly from 53.5% (38/71) in 2008 to 85.5% (47/55) in 2013 and 75% (3/4) in 2014 (average annual difference =  + 6.80%/year, OR: 1.43, 95% CI: 1.23–1.66, p < 0.001), and the percent of patients who reported heterosexual intercourses as a transmission route significantly decreased in this time period from 22.5% (16/71) to 3.6% (2/55) and 0% in 2014 (average annual difference = −5.10%/year, OR: 0.61, 95% CI: 0.49–0.77, p < 0.001), while the proportion of IDUs was stable (p = 0.340). Similar temporal trends for the mode of HIV-1 transmission were visible within the group of patients with LTHI, while the proportions of persons with the three transmission routes did not change significantly among patients with RHI (Table 6).

The proportion of non-R5 strains was stable over time with a slight decrease over the studied period (average annual difference = −1.52%/year, p = 0.234), and geno2pheno FPR values were also stable over time (p = 0.404). Similarly, there was a stable proportion of clustered HIV-1 sequences (p = 0.634), and the percent of infections with non-B subtypes over time (p = 0.445). The age at diagnosis was also stable throughout the examined time range (p = 0.108). The same stable trends were observed in the separate analyses restricted to the patients with RHI and LTHI, with the exception of a tendency to the less frequent clustered transmission through years among patients with RHI (p = 0.074) (Table 6).

## Discussion

In the studied group of 292 patients with HIV-1 infection diagnosed in the years 2008–2014 in four centers for HIV Diagnostics and Therapy for AIDS located in southern and central Poland (Chorzów, Kraków, Łódź, and Wrocław) recent HIV-1 infections accounted for 15.8% of all newly diagnosed infections. This is a much smaller proportion of recent infections than observed in Poland in 2006 with the BED assay (44%)^27^. Although the latter may have been overestimated due to the substantial false recent rate of the BED test, it also coincides with significant upsurge of infections among MSM, which occurred in the mid-2000^28^. Thus, the smaller, but gradually increasing, percent of recent infections observed in the current study likely represents a real feature of the epidemic. In the current study recent HIV-1 infection status was detected with quantitative limiting antigen avidity enzyme immunoassay (LAg-Avidity EIA), which provides the false recent rate as low as 1.3% (0.3–3.2)^29^. The accuracy of RHI assessment by the LAg-Avidity EIA seems to be supported by the significantly lower frequency of ambiguous nucleotides in *env* sequences obtained from individuals with RHI than in sequences from patients with LTHI, as the proportion of ambiguous nucleotides is known to increase with the stage of infection^30^.

The overall frequency of non-B subtypes in the examined group was 4.5% (13/292), and was comparable for patients with recent and long-term HIV-1 infection. Nevertheless, this value was considerably lower than the frequency of non-B clades determined in other Polish study covering similar time period (112/946; 11.8%; p < 0.001)^31^. The generally low frequency of non-B HIV-1 variants observed in our study may be explained by low frequency of non-B subtypes among samples obtained from patients diagnosed in Upper Silesia region (Chorzów, 2/130; 1.5%), which constituted the most prevalent group in our research, and were not included in the previous study. This finding supports the notion that the prevalence of non-B HIV-1 subtypes may differ by geographic region. Additionally, HIV-1 infections with sub-subtype A6 and CRF50_A1D which were previously reported to spread in former Soviet Union area and Western European countries, respectively^32,33^, were detected in Poland for the first time. Similarly to the previous study^31^, the proportion of non-B variants in our study was stable throughout the study period.

Admittedly, patients with HIV-1 diagnosis during long-term infection predominated in our study. However, we found both, a significant increase of the proportion of recent infections and an increase in CD4^+^ T-cell count among the new HIV diagnoses over the study period, from 2008 to 2014. These data suggest that during the study period the average time from infection to HIV diagnosis decreased, although this decrease was not spectacular and should be presumably measured in weeks or months rather than in years. Indeed, such a decline in the estimated mean interval of time-to-HIV-diagnosis from 4.0 in 2001 to 3.2 years in 2010 was previously reported for MSM in United Kingdom^34^, and a gradual decrease of time from infection to diagnosis was observed between 2012 and 2016 across the European Union^35^. It is also in line with the previously described increasing testing rates^36^, and higher rates of HIV-1 testing associated with the same-sex sexual exposure^37^.

The overall distribution of the transmission routes reported by patients with RHI and LTHI was comparable. However, there was a significant annual decrease in the proportion of women and individuals infected through heterosexual contact, coupled with an increase in proportion of MSM. These temporal trends for the HIV-1 transmission route were observed within the whole studied group and among patients with LTHI and RHI separately, only in the latter group not reaching the level of significance. Hence, these data may confirm increase in the frequency of MSM testing for HIV-1 infection, and indicate that the epidemic among MSM in Poland was on the rise, while the increasing testing rates were still not sufficient to achieve an HIV diagnosis within the first 6 months after infection in the majority of cases.

In the analysis of phylogenetic clusters, sequences from 15 (32.6%) out of 46 patients with RHI were included in 14 clusters. Similarly to other reports^38,39^, sequences from individuals with RHI were notably more frequent within the clusters than outside the clusters (26.3% *vs* 13.2%). This higher frequency may be explained by the fact that the samples obtained in early infection experience minimal genetic divergence and are more likely to cluster with the putative donor sequence^24^. Besides, the majority of clustering sequences obtained from patients with RHI (13/15) were grouped with the sequences derived from patients with LTHI. Considering that in most of these mixed clusters the date of sample collection for a patient with RHI was less than 6 months earlier than the sampling date for a patient with LTHI or the sample collection dates were the same for both patients, it may suggest that the patients with long-standing infection were presumably responsible for the virus propagation, with most of them likely transmitting the virus before being HIV-1-diagnosed, and thus being not aware of their HIV-1 status. Although such an assumption could be confirmed by the medical interview data only in the case of one pair of patients with RHI and LTHI, it is in line with the results of the country-wide survey performed among MSM in the Netherlands revealing that over 70% of transmission events in the years 1996–2010 originated from undiagnosed individuals^40^. On the other hand, the lack of clustering for 67.4% (31/46) of sequences from patients with RHI may indicate that a considerable proportion of HIV-1 infections could be transmitted by undiagnosed (or not linked to care) individuals. However, due to the relatively low sample size in our study, also individuals that were not sampled should be considered as a potential source of HIV-1 infection for patients with RHI.

The frequency of clustered HIV-1 sequences identified in our study (57/292, 19.5%) was within the range of the frequencies observed in other European and North American HIV-1-positive groups^38,41–45^. Similarly to other European and North American cohorts^38,41–46^ the proportion of MSM (together with men who reported sex with either men or women as a transmission route) was significantly higher among patients with clustered sequences compared to non-clustered (clustered: 48/57, 84.2% *vs* non-clustered: 164/235, 69.8%; p = 0.031), showing that, like in other regions, the epidemic in southern and central Poland has been driven mainly by MSM. However, according to our data, unlike in most other studies^38,41,43–46^, patients with clustered HIV sequences were not significantly younger than patients with viral sequences located outside the clusters.

According to our data, the proportion of all sequences clustering with other sequences was stable over time or tended to decrease during the study period among samples obtained from patients with RHI. This is opposite to what was observed in a prior study in Poland, where significantly increasing trends for clustered transmissions were reported^46^. However, we only collected samples from four of 16 regions in Poland. In our sample 4/27 clusters (14.8%) contained sequences from different regions. Such interregional clusters with highly related sequences coming from different regions were also found by other researchers in Poland^46^ and Germany^44^, and in comparison to our results the proportion of these clusters was shown to be higher (31/109, 28.4% and 32/184, 17.4%, respectively). It is possible that due to increasing mixing, other patients with clustering sequences were diagnosed in other regions of Poland, and therefore were not captured, explaining the lower than expected level of interregional clusters in our study. In line with other studies^44,46^, sequences from MSM were the most common in the interregional clusters (7/8, 87.5%), confirming the role of MSM in bridging regional HIV epidemics.

The overall prevalence of non-R5 strains in our study was 23% as detected with geno2pheno 10% FPR for subtype B samples and PhenoSeq for non-B subtypes or geno2pheno 10% FPR for all samples, and was slightly lower than non-R5 prevalence determined in the former study (28%) with the geno2pheno 10% FPR, among newly diagnosed patients from northern Poland^22^. This small discrepancy could have resulted from the diverse approach to tropism prediction in both studies, namely single *vs* triplicate sequencing of V3 coding region in the current and the previous study, respectively. Triplicate genotypic V3 testing is able detect more non-R5 variant in comparison with analysis of single sequences. However, tropism predictions using V3 genotyping based on single or triplicate testing using geno2pheno with a FPR of 10% were shown to be comparable, and the high concordance of tropism prediction among samples with single and triplicate amplification stands behind the use of single amplification in diagnostic practice^47–51^. Alternatively, the lower frequency of non-R5 viruses in our study may be explained by the local, within-country differences in the occurrence of CXCR4-using strains. This may be supported by the observed stable prevalence of non-R5 strains during the study period among all patients with new HIV-1 diagnoses and among patients with recent and long-term HIV-1 infection separately, whereas in previous study performed in northern Poland an increasing trend for the frequency of the non-R5 strains was reported among patients with new diagnoses^22^. It is also noteworthy that the median value of geno2pheno FPR in our study was relatively low (23.5%; (10.5–49.8%)), since it was demonstrated that low baseline FPR determined by the geno2pheno tool can predict tropism switch from CCR5 to CXCR4, and patients with R5 viruses predicted at diagnosis with a geno2pheno FPR of less than 50% (or <40.6%) were more prone to switch coreceptor over time than patients with FPR values of >50% (or >40.6)^17,52^.

The presence of non-R5 strains among persons with RHI may suggest possible transmission of non-R5 strains. In the current study, the frequency of non-R5 strains was comparable between groups of patients with RHI (24%) and LTHI (23%). Such a relatively high frequency of CXCR4-using strains among patients with RHI was not commonly observed in other groups of patients with similar characteristics (recent or acute HIV-1 infection, subtype B predominance, mainly sexual mode of transmission), and similar method of tropism prediction (geno2pheno with 10% FPR). For instance, according to genotypic tropism testing performed on proviral DNA in Italy only 3% of patients with RHI were infected with X4 strains^11^, and in other studies the frequency of CXCR4-using strains among patients at early infection stages was under 20%^5,12,15–17,53^. The relatively high frequency of non-R5 strains determined in the current study for patients with RHI deserves attention because of the restricted number of patients eligible to therapy with CCR5 antagonist, maraviroc, as well as in the light of the established correlation between infection with the CXCR4-using strains and faster disease progression^15,54^, or first-line treatment failure^19^.

Although in current and other studies^5,15,53,55,56^ there were no significant differences between patients with RHI harbouring non-R5 and R5 viruses with regard to the baseline CD4^+^ T-cell count and viral load, longitudinal observations indicated that the presence of CXCR4-utilizing strains at the beginning of infection was associated with faster disease progression characterised by accelerated CD4^+^ T-cell count decline below 350 cells/μl^5,53^. Besides, no significant differences in sex, age, CCR5 Δ32 genotype, and HIV-1 subtype between patients with possible transmission of non-R5 and R5 strains were found in our research, confirming the results obtained in other studies^15,53,57^.

In our study, non-R5 strains among patients with RHI were observed exclusively in MSM, while patients with all other routes of HIV-1 transmission were harbouring R5 viruses. In some surveys addressing the issue of putative transmission of X4 strains to the new hosts, the route of HIV-1 transmission was similar among patients with R5 and X4 variants^15,53,55,58^, whereas in others, X4 viruses were more frequently detected in IDUs than in patients infected by sexual contacts^5,56^. In our study the number of RHI attributed to the transmission routes other than sex between men was very small, thus our ability to study the differences by transmission route was limited. Nonetheless, our observations are consistent with prior research suggesting that proposed barriers protecting against X4 strains infection via blood and mucosal epithelium, and possibly selecting for R5 variants during transmission^8^ are similarly not perfect, and the spread of non-R5 and R5 strains may occur as a random process^57^.

Since detection of non-R5 strains among patients with RHI may reflect either the initial transmission of such strains or rapid evolution of X4 viruses from acquired R5 strains, to further address the issue of possible transmission of non-R5 strains to the new hosts among MSM, the analysis of transmission clusters identified by phylogenetic approach was performed. Among 11 patients with RHI and non-R5 strains, 5 had their HIV-1 sequences included within separate phylogenetic clusters indicating related transmission. Only in one of these 5 clusters HIV-1 sequences presented different tropism (non-R5 and R5). These clustered sequences were derived from 2 patients with RHI, however, the patient with R5 strain infection entered the study over 4 years earlier than the patient harbouring non-R5 variant. Thus, either he was not the immediate donor to the second patient, or he was not under successful treatment during 4 years after diagnosis. If the latter is true, it is possible that at the time of HIV-1 transmission, non-R5 variants could have emerged in the putative donor patient, initially infected with R5 virus, and could have been transmitted to a new host. Unfortunately, we cannot confirm this assumption, since we have neither an additional, later sample from the patient with R5 strain infection nor additional clinical information. In the remaining 4 clusters the second patient involved in the same cluster (putative donor) was a person with LTHI, also harbouring non-R5 strains, which may indicate the acquisition of non-R5 viruses, and the prevalence of acquired non-R5 HIV-1 infection confirmed by the phylogenetic analysis can be settled at 8.7% (4/46). Studies in which the possibility of transmission of non-R5 strains was evaluated using phylogenetic clusters analysis are scarce, and give inconsistent results. Frange *et al*.^58^ failed to confirm the transmission of non-R5 strains in the group of patients with primary HIV-1 infection, as only 1 out of 27 non-R5 sequences was present within a transmission cluster, and grouped with the sequence of an R5 strain obtained from a patient who was infected 34 months after infection of the presumed donor harbouring non-R5 virus. In turn, our data are congruent with the results presented by Chalmet *et al*.^57^. In their study transmission of non-R5 strains was suggested by the presence of non-R5 sequences within common phylogenetic clusters, and was confirmed by the identification of non-R5 strains in both partners of well-recognised transmission pairs shortly after infection, supporting the hypothesis of random spread of strains presenting non-R5 or R5 tropism.

The major limitation of our study is a relatively small sample size, hence we received small cluster sizes (mostly pairs) and some important transmission links could be omitted. Although patients included in the analysis constituted an estimated sample of 16% of all new diagnoses detected in the studied provinces during study period, and such a sample is expected to be representative of the examined area, the interpretation of the transmission clusters in such a case should be performed with the extreme caution. Especially, it should be considered that individuals who were not sampled can be either the intermediate hosts between patients with viral sequences gathered within the same cluster or the direct source of HIV-1 infection for them.

Another limitation of our study is linked to the years of samples collection, i.e. 2008–2014. While it is intrinsic to the similar types of studies that sampling considerably precedes the time of publication making the results significant for the earlier time periods^38,39,45,59^, we have tried to extend the validity of our study by conducting the time trends analyses.

Moreover, unlike in most other studies where *pol* HIV-1 sequences were used, we have used *env* gene sequences to detect phylogenetic transmission clusters, which complicates direct comparisons between studies. However, it was recently found that *env* sequences, due to their higher variability, may be more suitable than the *pol* sequences for analysis of recent infections^59^.

Finally, tropism predictions in our study were performed solely with the genotypic methods. Although genotypic tropism testing is currently accepted as an alternative to the phenotypic testing^47,60^, and >80% concordance between phenotypic assays and the geno2pheno method used for subtype B tropism assessment was reported^51,61–65^, it was shown that detection of CXCR4-using viruses by some genotypic assays may be inaccurate, especially for non-B subtypes^61,66,67^. Thus, to predict the tropism in non-B HIV-1 samples we used PhenoSeq, a tool with improved sensitivity and specificity for establishing the tropism of non-B subtypes^68^. Nevertheless, the discrepant results of the genotypic and phenotypic tropism assays were reported for patients with acute or recent infections^5,13,14,16^, indicating that our tropism predictions should be interpreted with caution.

In conclusion, we found relatively high prevalence of non-R5 strains among Polish patients with new HIV-1 diagnoses (23%) and recent infections (24%), but we did not confirm the increasing trend for the frequency of non-R5 viruses among newly diagnosed individuals reported in a former Polish study. Transmission of non-R5 viruses was confirmed by cluster analysis for 8.7% (4/46) patients with RHI. Non-R5 strain distribution and recent HIV-1 infection frequency, as well as the prevalence of non-B subtypes in Poland may differ by geographic region. Although viral sequences obtained from individuals with RHI were notably more frequent within the transmission clusters than outside them, the participation of these individuals in the forward HIV-1 transmission was not confirmed.

## Methods

### Study group and sample collection

The study was performed among 298 consecutive Polish patients who were recruited under the following inclusion criteria: (1) being newly diagnosed with HIV-1 infection, and having no clinical AIDS (indicator disease) at first testing; (2) presenting at 1 of 4 centers for HIV Diagnostics and Therapy for AIDS in Poland placed in Chorzów, Kraków, Łódź, and Wrocław, during the enrolment period, between March 2008 and February 2014; (3) having their blood sample collected at first presentation; and (4) providing written informed consent to participate in the study. During the recruitment procedure the data regarding sex, age, date and city of HIV-1 diagnosis, as well as self-reported transmission route were collected. Viral load and CD4^+^ T-lymphocyte at HIV-1 diagnosis were retrieved from patients’ medical records and were the first results obtained after HIV-1 diagnosis. Anticoagulated venous blood and plasma samples were collected at first clinical presentation and were stored at –80 °C until further laboratory procedures were performed.

All plasma samples were tested with the quantitative limiting antigen avidity enzyme immunoassay (Sedia^TM^ HIV-1 LAg-Avidity EIA, Sedia BioSciences Corporation, Portland, Oregon, USA) to differentiate recent from long-term HIV-1 infections. Assay was performed according to the manufacturer’s protocol^69^. As recommended, samples with normalized median optical density values (ODn) ≤ 1.5 in a triplicate confirmatory testing were considered to be obtained from persons with recent HIV-1 infection, which corresponds to a duration of HIV-1 infection no longer than 130 days (95% CI: 118–142) since seroconversion^70^.

QIAamp DNA Blood Mini Kit (QIAGEN GmbH, Hilden, Germany) was used to extract genomic and proviral DNA from blood samples. The presence of 32 base-pair deletion in patients’ CCR5 gene (Δ32) was determined with the polymerase chain reaction (PCR) as described previously^10,71^.

### Amplification and sequencing of HIV-1 *env* gene

Amplification of *env* gene fragments coding for gp120 region was performed with nested-PCR in 298 samples of genomic and proviral DNA obtained from 47 patients with recent HIV-1 infection (RHI), and 251 persons with long-term infection (LTHI). In the first step of the nested-PCR, fragment spanning nucleotides 6201–9089 was amplified using E00-F and E01-R as outer primers^72^, with the following amplification conditions: an initial denaturation at 94 °C/7 minutes, followed by 40 cycles of 94 °C/40 seconds, 51 °C/40 seconds, and 72 °C/3 minutes, with the final extension at 72 °C/7 minutes, in a final volume of 50 μl. In the next step, inner primers ED5-F and E125-R were used to obtain 782 base pairs fragment of gp120 spanning nucleotides 6557-7338^72,73^. Amplification conditions for inner primer pairs were as follows: 94 °C/5 minutes, followed by 35 cycles of 94 °C/35 seconds, 55 °C/35 seconds, and 72 °C/90 seconds, with the final extension at 72 °C/5 minutes, in a final volume of 50 μl.

Purified nested-PCR products were subjected to the population-based sequencing with the ABI Prism Big Dye Terminator v3.1 cycle sequencing kit (Applied Biosystems, Foster City, CA, USA) with the primers used in the inner step of nested-PCR. For each sample, the sequences of both strands were determined separately using 96-capillary 3730xl DNA Analyzer (Applied Biosystems, USA). The obtained sequences were manually checked and trimmed to remove primers, resulting in fragments spanning nucleotides from 6583 to 7314 corresponding to codons 121–363 of gp120, covering the complete coding sequence for V1, V2, C2 and V3 regions. Nucleotides were considered ambiguous, when the next highest peak in the electropherogram exceeded 25% of the main peak. All nucleotide positions in the manuscript are presented according to the numbering positions of HIV-1 HXB2 (GenBank accession number: K03455). Amplification and sequencing was successful for 292 specimens, including 46 originated from patients with RHI.

### Analysis of HIV-1 *env* sequences and coreceptor usage prediction

All sequences were analysed for the presence of hypermutations using HYPERMUT software v2.0^74^. HIV-1 subtypes were initially determined with REGA HIV-1 Subtyping Tool 3.0 (http://dbpartners.stanford.edu:8080/RegaSubtyping/stanford-hiv/typingtool), and afterward with NCBI Genotyping Tool using HIV-1 reference set from 2009 (https://www.ncbi.nlm.nih.gov/projects/genotyping/formpage.cgi). The NCBI Genotyping Tool analyses were performed especially to check sequences determined as non-B or not assigned to any subtype by REGA Tool. To confirm subtype identification by REGA and NCBI genotyping tools, phylogenetic analysis with PhyML 3.0 online software was conducted^75^. Reference HIV-1 sequences of known genotype (subtypes and circulating recombinant forms (CRFs)), that were used in phylogenetic analysis, were retrieved from Los Alamos National Laboratory HIV Sequence Database’s compendium from the year 2016 (https://www.hiv.lanl.gov/content/sequence/NEWALIGN/align.html). Phylogenetic tree for all examined and reference sequences was created in the PhyML 3.0 with the maximum likelihood (ML) method under the general time reversible nucleotide substitution model + gamma distribution of rates + proportion of invariable sites (GTR + G + I) with the gamma shape parameter and proportion of invariable sites estimated from data (Supplementary Fig. S1). Tree topology was improved with both NNI (Nearest Neighbor Interchange) and SPR (Subtree Pruning and Regrafting) algorithms. Subsequently, all examined sequences were evaluated for recombination with the jumping profile hidden Markov model (jpHMM, http://jphmm.gobics.de/submission_hiv)^76^.

To determine HIV-1 coreceptor usage, all single proviral DNA *env* sequences coding for a V3 loop were analysed with the online geno2pheno algorithm (http://coreceptor.geno2pheno.org/index.php)^77^, which is one of the most widely accepted and used methods for genotypic prediction of viral tropism. In accordance with the current recommendations^47^ and results of prospective studies^11,12,78^, a false positive rate (FPR) of 10% was used to interpret the clonal geno2pheno’s results, thus viruses were classified as non-R5 when V3 sequences displayed an FPR value ≤ 10%, (10% probability of classifying an R5 virus falsely as X4). Since the sensitivity of the geno2pheno method to detect X4 variants was shown to be lower for non-B subtypes than for subtype B^67^, sequences representing subtypes other than B were tested with PhenoSeq program, which is a newer tool developed to reliably predict the tropism of HIV-1 variants such as A, B, C, D, CRF01_AE and CRF02_AG (http://tools.burnet.edu.au/phenoseq/)^68^.

Geno2pheno algorithm with FPR of 5.75 was also used to improve the specificity, and higher FPR values (of 15% and 20%) were applied to increase the sensitivity for detection of non-R5 variants.

The number of ambiguous nucleotides in the obtained V3 coding fragments ranged from 0 to 7, thus all 292 V3 sequences could be included in the genotypic tropism predictions, since it is not recommended to consider the V3 sequences with ≥8 ambiguous nucleotides in a coreceptor usage testing^47^.

### Phylogenetic analyses and identification of clustered HIV-1 *env* sequences

Prior to phylogenetic analyses ClustalX algorithm was used to align 292 *env* sequences together with group M consensus sequence^79^. The alignment was manually edited using the BioEdit program^80^, and columns with gaps were removed. The GTR + G + I was selected as the best fitting nucleotide substitution model according to the Akaike and Bayesian Information Criterions (AIC, BIC) implemented in the jModeltest 2.1.9 software^81^. Under this model the nucleotide frequencies were: A = 0.4243, C = 0.1988, G = 0.1723, T = 0.2046; the substitution rates were: AC = 1.2416, AG = 3.9991, AT = 0.7674, CG = 0.5556, CT = 3.7929, GT = 1.0000; proportion of invariant = 0.165; and gamma shape parameter = 0.776.

Initially, transmission clusters were investigated with the PhyML 3.0 software^75^ using maximum likelihood (ML) method, under the selected GTR + G + I model, with the best of NNI and SPR algorithms for tree topology improvement. To evaluate branch supports of a phylogeny approximate likelihood ratio test (aLRT) values with nonparametric Shimodaira–Hasegawa–like (SH–like) algorithm were computed in the PhyML 3.0. The resulting ML phylogenetic tree and aligned sequence data were analysed with the Cluster Picker 1.2.5 software to identify monophyletic clusters based on specified thresholds for maximum pairwise genetic distances and aLRT support values^82^.

Additionally, a more robust Bayesian Metropolis coupled Markov chain Monte Carlo (MCMCMC) method was used to further verify the clusters. Two independent replicates of 50 million generations were run in MrBayes v.3.2.6^83^ with sample frequency of 2500, under GTR + G + I model. A burn-in of 25% was used to summarize parameters and trees in the Bayesian approach. To control whether a sample from the posterior probability distribution was adequate, a plot of the generation *vs* the log probability was checked. The total effective sample size (ESS) values were above 200 for all parameters, average standard deviation of split frequencies decreased below 0.01 after 11.9 million generations, and potential scale reduction factors (PSRFs) were reasonably close to 1.0 indicating appropriate convergence. Finally, transmission clusters were identified when the following criteria were simultaneously met: i) aLRT value in ML method >0.9, ii) maximum within cluster pairwise genetic distance calculated in the Cluster Picker <3%, and iii) posterior probability in a Bayesian inference = 1. Such clusters are assumed to represent related transmission events between patients whose viral sequences are gathered within a single cluster. Phylogenetic trees were visualized in FigTree v.1.4.3.

### Sequence data

All of the HIV-1 nucleotide sequences obtained in the study have been deposited in GenBank with the KT778123-KT778247 and MH627052-MH627218 accession numbers.

### Statistical analysis

Comparisons of categorical variables between specified groups were performed with the Pearson’s Chi-square test. Two-tailed Fisher’s exact test was applied for binary variables and for variables with several possible categories to compare a specified category *vs* all other categories. The nonparametric Mann-Whitney *U* test was used for analysis of continuous variables. To examine the time trends in selected parameters, logistic and linear regression analyses with the year of HIV-1 diagnosis as a single predictor were performed for binary and continuous outcome variables, respectively. For the continuous outcome variables we present the slope of the linear effect of the diagnosis year. For the binary variables odds ratios per each additional year are provided. For greater comparability, for the binary variables we also calculated average prevalence differences per year (average annual difference), by fitting linear regression model to annual average prevalences. All statistical analyses were performed using STATISTICA v13.1 enriched with a Medical Set (Statsoft, Warsaw, Poland), with the significance level defined by P < 0.05.

### Ethical approval

The research was approved by the National Institute of Public Health – National Institute of Hygiene Bioethics Committee, Poland (no. 3/2007), and all procedures were performed in accordance with relevant guidelines and regulations. All collected samples and data were anonymous and coded. Written informed consent was obtained from study participants.

## Supplementary information

Supplementary information
