# Supplementary material for: Transmission patterns of HIV-1 non-R5 strains in Poland
Source: Sci Rep. 2019 Mar 21;9:4970. doi: 10.1038/s41598-019-41407-7 (PMC6428829; doi:10.1038/s41598-019-41407-7)
Supplement: Supplementary file 1 — Supplementary information [file 41598_2019_41407_MOESM1_ESM.pdf]

Transmission patterns of HIV-1 non-R5 strains in Poland

Joanna Smoleń-Dzirba[1], Magdalena Rosińska[2], Piotr Kruszyński[1], Janusz Janiec[2], Mariusz Cycoń[1], Jolanta Bratosiewicz-Wąsik[3], Marek Beniowski[4], Monika Bociąga-Jasik[5], Elżbieta Jabłonowska[6], Bartosz Szetela[7], Tomasz J. Wąsik[1,*]

[1]*Department of Microbiology and Virology, School of Pharmacy with the Division of Laboratory Medicine in Sosnowiec, Medical University of Silesia, Katowice, Poland,*

[2]*Department of Infectious Disease Epidemiology and Surveillance, National Institute of Public Health - National Institute of Hygiene, Chocimska 24, Warsaw, Poland,*

[3]*School of Pharmacy with the Division of Laboratory Medicine in Sosnowiec, Medical University of Silesia, Katowice, Poland, Department of Biopharmacy,*

[4]*Out Patients Clinic for AIDS Diagnostics and Therapy, Specialistic Hospital in Chorzow, Zjednoczenia 10, Chorzów, Poland,*

[5]*Department of Infectious Diseases, Jagiellonian University Medical College, Śniadeckich 5, Kraków, Poland,*

[6] *Department of Infectious Diseases and Hepatology, Medical University of Lodz, Kniaziewicza 1, Łódź, Poland,*

[7]*Department of Infectious Diseases, Hepatology and Acquired Immune Deficiencies, Wroclaw Medical University, Koszarowa 5, Wrocław, Poland*

*Address correspondence to this author at the School of Pharmacy with the Division of Laboratory Medicine in Sosnowiec, Medical University of Silesia, Katowice, Poland, Department of Microbiology and Virology, ul. Jagiellońska 4, 41-200 Sosnowiec, Poland, Tel: +48 32 364 16 21, Fax: +48 32 364 15 02, E-mail: twasik@sum.edu.pl

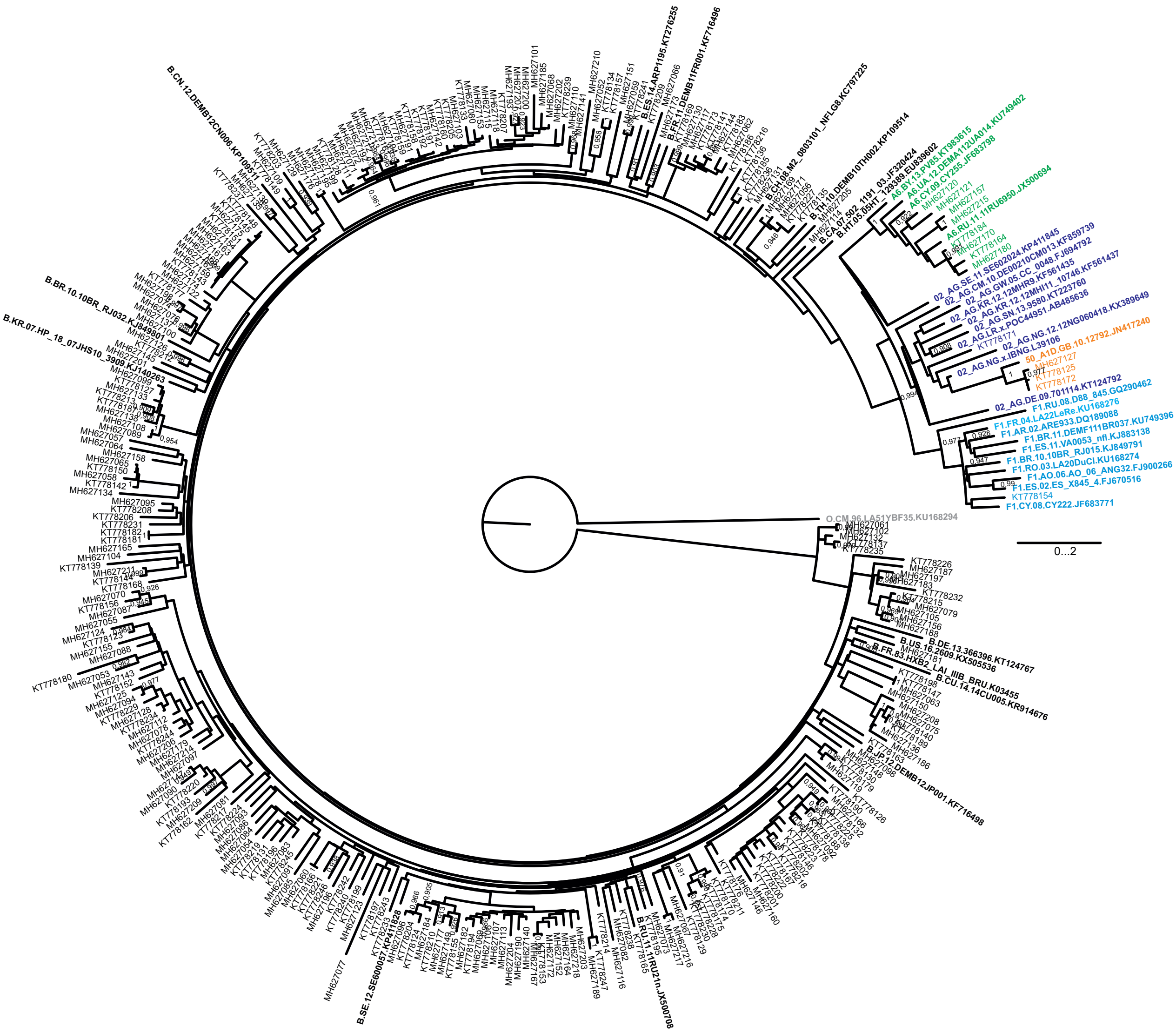

Figure S1. Determination of HIV-1 subtypes and circulating recombinant forms (CRFs) with the phylogenetic analysis. Maximum likelihood (ML) phylogenetic tree constructed for *env* sequences obtained from 292 patients diagnosed in Poland in the years 2008-2014 together with the reference HIV-1 sequences of known genotype (subtypes and CRFs), retrieved from Los Alamos National Laboratory HIV Sequence Database's compendium from the year 2016 (https://www.hiv.lanl.gov/content/sequence/NEWALIGN/align.html). The phylogenetic tree was rooted with the group O reference sequence. For a better clarity, only the reference sequences for HIV-1 subtypes and CRFs with which the sampled sequences were found to cluster (in bold), and aLRT values of >90% were presented on the ML tree. Each subtype and CRF is marked with a different colour.
